# Paradoxical Association of Postoperative Plasma Sphingosine-1-Phosphate with Breast Cancer Aggressiveness and Chemotherapy

**DOI:** 10.1155/2017/5984819

**Published:** 2017-09-24

**Authors:** Rajesh Ramanathan, Ali Raza, Jamie Sturgill, Debra Lyon, Jessica Young, Nitai C. Hait, Kazuaki Takabe

**Affiliations:** ^1^Department of Surgery, Virginia Commonwealth University Medical Center, 1200 E. Broad St., Richmond, VA, USA; ^2^Lincoln Medical and Mental Health Center, Cancer Center, Room 9-69, Bronx, NY, USA; ^3^Department of Family and Community Health Nursing, Virginia Commonwealth University, 1100 E. Leigh St., Richmond, VA, USA; ^4^Department of Molecular and Cellular Biology, Roswell Park Cancer Institute, Buffalo, NY, USA; ^5^University of Florida, College of Nursing, Gainesville, FL, USA; ^6^Breast Surgery, Department of Surgical Oncology, Roswell Park Cancer Institute, Buffalo, NY, USA; ^7^Department of Surgery, University at Buffalo Jacobs School of Medicine and Biomedical Sciences, The State University of New York, Buffalo, NY, USA

## Abstract

Sphingosine-1-phosphate (S1P) is a bioactive lipid mediator that has been shown to serve an important regulatory function in breast cancer progression. This study analyzes plasma S1P levels in breast cancer patients undergoing adjuvant therapy as compared to healthy control volunteers. 452 plasma S1P samples among 158 breast cancer patients, along with 20 healthy control volunteers, were analyzed. Mean S1P levels did not significantly differ between cancer patients and controls. Smoking was associated with higher S1P levels in cancer patients. Baseline S1P levels had weak inverse correlation with levels of the inflammatory mediator interleukin- (IL-) 17 and CCL-2 and positive correlation with tumor necrosis factor alpha (TNF-*α*). Midpoint S1P levels during adjuvant therapy were lower than baseline, with near return to baseline after completion, indicating a relationship between chemotherapy and circulating S1P. While stage of disease did not correlate with plasma S1P levels, they were lower among patients with Her2-enriched and triple-negative breast cancer as compared to luminal-type breast cancer. Plasma S1P levels are paradoxically suppressed in aggressive breast cancer and during adjuvant chemotherapy, which raises the possibility that postoperative plasma S1P levels do not reflect S1P secretion from resected breast cancer.

## 1. Introduction

Breast cancer is the second leading cause of cancer death among women in the United States with 40,610 deaths estimated in 2017 [1]. Local control of breast cancer by surgical resection and radiation, followed by adjuvant systemic therapy with chemotherapy, hormonal and targeted therapy, constitutes the mainstay of treatment for the majority of patients with breast cancer. However, given that the majority of deaths by breast cancer are due to distant recurrences and metastases, adjuvant therapy has been shown in numerous randomized trials to improve survival outcomes by addressing occult cancer cells using systemic therapy in the postoperative period. Unlike in the neoadjuvant setting, where the effect of therapy on tumor size is radiographically measurable, evaluation of the effect of adjuvant therapy is challenging. To date, there is no established biomarker to evaluate the effect of adjuvant systemic therapy during treatment, and the harsh reality is that its effectiveness of adjuvant therapy is only realized when there is clinical or radiographic recurrence of cancer. Thus, a biomarker that reflects the effect of adjuvant therapy is expected to have remarkable impact on survival since we can expect to use it as a guide to tailor therapy through follow-up plans and use of alternative adjuvant therapy.

Most investigations on biomarkers have centered on measuring circulating tumor cells, proteins, and nucleic acids released from the breast tumor or cancer cells. More recently, however, the roles of sphingolipid mediators in breast cancer have been increasingly investigated due to recent advances in mass spectrometry [2]. One lipid mediator of increased interest is the ceramide-sphingosine pathway involving sphingosine-1-phosphate (S1P) [3]. S1P has been shown to be involved in cancer cell growth, cancer progression, and metastasis [4–8]. Our group has established technology to quantify S1P in the tumor interstitial fluid [9] and lymphatic fluid [10] and published that S1P promotes both angiogenesis and lymphangiogenesis [11, 12]. Further, we have found that S1P is increased in human breast cancer as compared to normal tissues [13] and that tumor S1P levels are significantly higher in patients with lymph node metastasis [14], which suggests that breast cancer cells secrete S1P into the tumor microenvironment and into the circulation. Recently, we found that S1P signaling plays an even more important role in metastatic triple-negative breast cancers [15]. We have also reported that S1P is involved in inflammation [16–19] and found that S1P links inflammation and cancer progression [20, 21]. Even though adjuvant chemotherapies are known to evoke inflammation, the variation and modulation of S1P during these adjuvant therapies have yet to be investigated. Such data is expected to provide additional insight into the use of S1P as a prognostic biomarker during adjuvant therapy.

In this prospective study among women with breast cancer, the association of circulating plasma S1P with demographic factors and during adjuvant therapy is investigated.

## 2. Materials and Methods

### 2.1. Sample Collection

All the patient and volunteer samples were collected with Virginia Commonwealth University and Massey Cancer Center Institutional Review Board (IRB) approval. Venipuncture plasma samples were collected from 158 women who underwent surgical resection of their breast cancer tumors. Paired samples were collected two weeks prior to adjuvant therapy (baseline), prior to the fourth cycle of chemotherapy (midpoint), and two weeks after completion of adjuvant therapy (completion). For a control sample, a one-time plasma sample was collected from 20 healthy women volunteers. The samples were snap-frozen and stored at −80°C.

### 2.2. Biochemical Analyses

Circulating S1P concentration was quantified using liquid chromatography-electrospray ionization tandem mass spectrometry (LC-ESIMS/MC) at the Virginia Commonwealth University Lipidomics Core as previously described [13, 14]. Briefly, this consisted of internal standards from Avanti Polar Lipids (Alabaster, AL), lipid extraction in 20 *μ*L aliquot of ethanol : methanol : water (7 : 2 : 1), and analysis for S1P using LC-ESIMS/MC.

Additionally, levels of various circulating inflammatory cytokines were measured from baseline serum samples with enzyme-linked immunosorbent assays. The cytokines measured included interleukin- (IL-) 1, IL-2, IL-4, IL-5, IL-6, IL-7, IL-8, IL-10, IL-12, IL-13, IL-17, G-CSF, GM-CSF, interferon gamma (IFN-*γ*), MCP-1, MIP-1, and tumor necrosis factor alpha (TNF-*α*).

### 2.3. Statistical Analyses

With IRB approval, patient demographics, treatments, and pathology results were correlated with S1P levels at the various times points and compared with the control values. Paired and unpaired *t*-tests, chi-square analyses, and ANOVA analyses were used to perform the statistical analyses with statistical significance at *p* < 0.05.

## 3. Results

### 3.1. Circulating S1P Does Not Differ Significantly between Healthy Controls and Postoperative Breast Cancer Patients

There were 452 plasma S1P samples collected from the 158 breast cancer patients, along with 20 plasma S1P samples from the healthy control volunteers. Overall, there were no statistically significant differences in patient characteristics, such as age, body mass index (BMI), or ethnicity distribution between the breast cancer cohort and the control cohort (Table 1). Among the breast cancer patients, the mean age was 51.2 ± 9.8 years and BMI was 30.3 ± 7.6 kg/m^2^. The majority of the patients were Caucasian (58.2%) followed by African American (33.5%) and Hispanic (3.8%). There was no difference in plasma S1P level between the breast cancer patients at baseline and the control cohort (1221.7 versus 1139 pmol/mL, *p* = 0.41) (Table 1).

Of the breast cancer patients, a matched cohort with the controls was subselected matching for age (within five years), BMI (within five kg/m^2^), and ethnicity of the control patients. This resulted in 40 matched breast cancer patients by age, BMI, and ethnicity to the control volunteers. Similar to the overall analysis, there was no difference in baseline S1P level between the control-matched breast cancer patients and the controls (1260.9 versus 1139.1 pmol/mL, *p* = 0.19).

### 3.2. Circulating S1P Does Not Correlate with Age, BMI, Ethnicity, or Menstrual Status, but Does Correlate with Smoking among Breast Cancer Patients

Among the breast cancer cohort, there was no significant correlation between age and S1P concentration (*r*^2^ = 0.06, *p* = 0.32). There was no significant correlation between plasma S1P levels and BMI (*p* = 0.75) and plasma S1P and ethnicity (*p* = 0.41) in both the breast cancer and control cohorts. Among the breast cancer cohort, there was no significant difference in mean baseline plasma S1P levels between premenopausal patients and postmenopausal patients (1163.6 versus 1266.9 pmol/mL, *p* = 0.13). Tobacco use, however, was associated with increased plasma S1P levels (1445.4 versus 1163.3 pmol/mL, *p* < 0.01) among the breast cancer cohort, while alcohol use was not associated with S1P levels (*p* = 0.98).

### 3.3. Circulating S1P Is Inversely Associated with Tumor Grade and Aggressiveness

**Figure 1:**
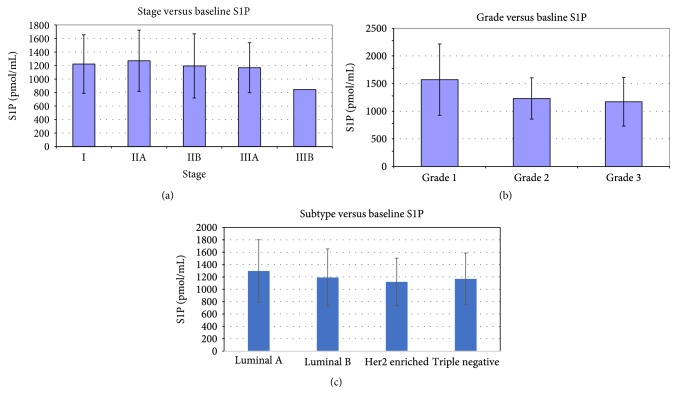
Association of circulating S1P with (a) breast cancer stage, (b) grade, and (c) histologic subtype.

Analyses of S1P levels and tumor grade and characteristics were evaluated among the breast cancer cohort. While stage of breast cancer was not associated with baseline S1P levels (*p* = 0.688), subtype and tumor grade were associated with baseline S1P (Figure 1()). Baseline S1P concentration was lower among patients with Her2-enriched and triple-negative breast cancer as compared to luminal-type cancer (1119.2 and 1167.1 versus 1280.8 pmol/mL, *p* < 0.05, Figure 1(c)). Additionally, patients with intermediate-grade and high-grade tumors similarly had lower S1P than those with low-grade tumors (1230.0 and 1176.5 versus 1570.8 pmol/mL, *p* < 0.05, Figure 1(b)). Although the circulating S1P was measured in the samples taken from patients in whom tumors were completely removed, this result appears somewhat paradoxical to the previous experimental studies suggesting a relationship between higher S1P and more aggressive cancer biology. On the other hand, our findings that circulating S1P levels were lower in Her2-enriched and triple-negative breast cancer patients are consistent with previous publications identifying that breast tumor S1P levels were lower in those patients with aggressive tumors [14].

### 3.4. Chemotherapy Is Associated with Changes in Circulating S1P

**Figure 2:**
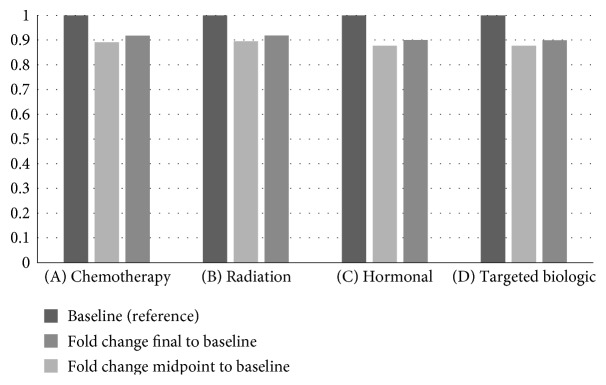
Change in S1P during (A) chemotherapy, (B) radiation therapy, (C) hormonal therapy, and (D) targeted biologic therapy.

In the paired sample analysis, for patient undergoing chemotherapy, the mean midpoint S1P concentration was significantly lower than the baseline and final concentration (1088.8 versus 1221.8 and 1121.5 pmol/mL, *p* < 0.01), suggesting an effect of chemotherapy on circulating S1P (Figure 2, A). Notably, there was no significant difference between baseline and final concentration (1221.8 versus 1121.5 pmol/mL, *p* = 0.06). Additionally, there were no significant differences in S1P concentration among patients who underwent radiation therapy (Figure 2, B), hormonal therapy (Figure 2, C), and targeted biologic therapy with trastuzumab (Figure 2, D).

**Figure 3:**
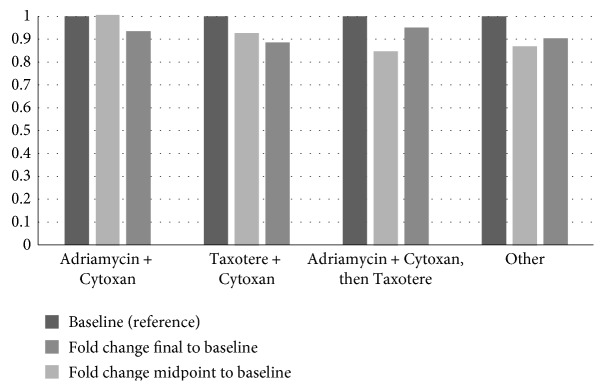
Change in S1P based on chemotherapeutic combination.

When assessing a type of chemotherapy, it did appear that S1P appeared to increase with AC therapy and remain high after completion, while it appeared to decrease with taxane-based chemotherapies (Figure 3).

### 3.5. Circulating Baseline S1P Levels Correlated with IL-17, CCL-2, and TNF-*α* Levels

Among the breast cancer cohort, associations between baseline plasma S1P levels and various inflammatory mediators were analyzed using Spearman's Rho tests. Baseline S1P had a significant weak inverse correlation with IL-17 (*σ* = −0.221, *p* = 0.045) and CCL-2 (*σ* = −0.317, *p* < 0.01), while it had a positive correlation with TNF-*α* (*σ* = 225, *p* = 0.016). Baseline plasma S1P did not have a significant correlation with the other measured inflammatory mediators.

## 4. Discussion

This study revealed that there was no statistically significant difference in circulating S1P levels between healthy volunteers and patients with breast cancer after tumor excision. The only demographic characteristic associated with increased circulating S1P was a history of smoking. Patients with more aggressive pathologic subtypes of breast cancer paradoxically demonstrated decreased levels of circulating S1P. While radiation, hormonal therapy, and targeted biologic adjuvant therapy did not affect S1P levels, chemotherapy was associated with changes in circulating S1P during treatment.

S1P itself results from the intracellular phosphorylation of sphingosine by two sphingosine kinases, SphK1 and SphK2 [3]. Phosphorylation of sphingosine by SphK1 allows the extracellular export of S1P [2]. Extracellular S1P binds to S1P cell surface receptors (S1PR1-5) in a multitude of cells and exerts its effects in an autocrine and/or paracrine manner. This interaction has been termed “inside-out signaling” [4].

The role of S1P in inflammation and immunity has been well described [16]. T cells, B cells, and endothelial cells all demonstrate unique profiles of S1P receptors (S1PR). These unique profiles influence and regulate development, recirculation, tissue homing patterns, and chemotactic responses in inflammation and immunity. S1PR have been shown to modulate monocyte activity through regulation of CD40 and TNF alpha [22–24]. Additionally, the S1P-S1PR1 axis is involved in lymphocyte trafficking leading to retention in inflamed tissue through the maintenance of S1P gradients [16]. In this study, we noted a positive correlation between baseline plasma S1P and TNF-*α*. Further investigation is needed to clarify the inverse relationship noted in this study between S1P levels and IL-17 and CCL-2 levels.

Clinically, S1P levels have been shown to be high in patients with breast cancer [13], and phosphorylated SphK1 levels are associated with high S1P levels in breast tumors [14]. S1P exerts its actions in cell survival migration and angiogenesis, thereby affecting cancer progression in several solid tumors [8]. We have reported that secreted S1P generates lymphatic vessels in tumor microenvironment [11, 12] and that is associated with increased lymph node metastasis in breast cancer patients [11, 14].

We have previously demonstrated that among patients undergoing total or partial mastectomy for tumors greater 1.5 cm, peritumoral tissues had significantly higher S1P and other sphingolipid levels as compared to distant tissues in the same sample [13]. In the present study, however, we did not identify increased circulating S1P in patients with breast cancer as compared to controls. Similarly, in the present study, there was no difference in circulating S1P by stage of disease. This was maybe because the breast tumors were already removed 2 weeks prior to collection of the blood samples in our cohort, and thus the effect of the tumor microenvironment was no longer there.

Further, higher grade and more aggressive subtypes such as Her2-enriched and triple-negative breast cancer patients demonstrated lower baseline circulating S1P levels. The lower baseline S1P levels in triple-negative breast cancer may be related to S1P's association with circulating estrogen [25, 26]. In studies among healthy volunteers, S1P levels were found to be higher in premenopausal women as compared to postmenopausal women and higher in premenopausal women as compared to men [25]. This effect was described in animal models due to increased S1P synthesis by estradiol through the activation of SphK1. These observations are in agreement with our previous report that breast cancer cells secrete S1P by estrogen stimulation [2].

Our findings of lower baseline S1P in a more aggressive disease run counter to the previous published data demonstrating an increase in tissue S1P signaling in aggressive tumors [27], but are consistent with the previous report that measured circulating S1P levels [14]. It is generally understood that activation of S1P pathway signaling is associated with a more aggressive disease. This paradoxical finding in our study could arise from the fact that studies indicating an association between S1P and tumor aggressiveness have been primarily tissue-based, thus reflecting the tumor microenvironment. Our study, however, measured circulating plasma S1P in patients after surgical excision of their tumors. We speculate that S1P is intrinsically involved in the local tumor microenvironment and exerts its actions locally as opposed to systemically, with no overall differences in circulating S1P. Thus, after surgical excision, the baseline S1P is likely no different from the controls due to the removal of the tumor microenvironment.

In support of that hypothesis is the finding that smoking was the only patient characteristic noted to result in increased circulating S1P. Smoking is known to result in systemic inflammation, and S1P's role in inflammation has been well described [28]. Specifically, the S1P pathway is involved in TNF-*α* signaling, NF-*κ*B activation pathways, and chemokine expression for recruitment of mononuclear cells into sites for inflammation [13, 29, 30]. As previously noted, our study did identify a positive correlation between S1P levels and TNF-*α* expression. The overall increased inflammatory state present in smokers likely explains the increased circulating S1P that was found in the present study. Another possibility is that smoking is known to be associated with dysfunction of the vessels, where S1P signaling plays fundamental roles. For instance, S1PR1-deficient mice die in utero from massive hemorrhage due to immature vessel development, and neutralization of extracellular S1P with anti-S1P antibody demonstrates a significant inhibition of angiogenesis, tumor growth, and metastasis [5]. Therefore, it will not be surprising if smoking causes a vasculopathy that induces S1P production and angiogenesis.

Adjuvant therapies in breast cancer aim to decrease both local and distant recurrence and/or metastasis from nodal spread or microscopic dissemination. Adjuvant treatment options include various methods of irradiation, multiple chemotherapy regimens, use of hormonal therapy, and biologic modulators. While radiation therapy is focused on the breast tissue field and hormonal therapy targets specific receptors in the breast and ovaries, the effects of chemotherapy are more systemic. Perhaps, this study identified no change in S1P during radiation therapy and hormonal therapy, however, did find changes in S1P with chemotherapy. Specifically, with AC chemotherapy, circulating S1P increased from baseline and remained high, while with taxane-based chemotherapies, S1P appeared to decrease. This may be related to the fact that doxorubicin in AC regimen induces inflammation.

The interaction between the S1P pathway and adjuvant treatment agents is an area of active investigation. Upregulation of the S1P pathway has been implicated in the development of chemotherapy resistance, whereby the addition of FTY720, a functional antagonist of S1PR1 that blocks S1P signaling, results in overcoming chemotherapy resistance [31, 32]. The mechanism for overcoming resistance may be related to ABC transporter upregulation as noted in S1P-lyase- (an enzyme that degrades S1P) deficient fibroblasts, which demonstrate increased doxorubicin resistance in B cell lymphoma [33]. Additionally, FTY720, through its inhibition of the S1P signaling, has been shown to inhibit the hypoxia-inducible factor (HIF) pathways and has been associated with increased sensitivity to gemcitabine-based chemotherapy in renal cell cancer and docetaxel in prostate cancer [34, 35].

Specifically, in breast cancer, there have been a limited number of studies investigating the interaction between S1P and chemotherapy. The only study to date investigating circulating S1P, to the authors' best knowledge, details the detrimental effect of circulating S1P with weight gain during chemotherapy among 21 patients [36]. In breast cancer cell lines, however, the use of tamoxifen and medroxyprogesterone result in the downregulation of S1PR3 and stimulation of S1PR2 with activation of autophagy of the breast cancer cells towards death, again demonstrating the detrimental role of S1P pathway activation in cancer [30]. The present study adds additional data that suggest not only a role of S1P in modulating the effects of chemotherapy but also the effect of chemotherapy on S1P levels. It stands to reason that the decrease in S1P levels associated with taxane-based therapies would be beneficial for those patients, while the increase in circulation S1P with AC chemotherapy would likely be detrimental.

In breast cancer cells, SphK1 activity has been linked to endocrine resistance, whereby overexpression of SphK1 resulted in resistance to tamoxifen with increased proliferation [26]. Tamoxifen-resistant cells displayed increased SphK1 activity. Inhibition of SphK1 resulted in restoration of tamoxifen's activity, thus demonstrating the role of the SphK1 pathway in estrogen receptivity and tamoxifen resistance.

The main limitations of this study include the limited patient sample, although to the authors' best knowledge, this constitutes the largest study investigating the association of circulating S1P with patient characteristics and during chemotherapy. The study also used a limited control subset of patients without breast cancer to investigate differences in baseline S1P between controls and postoperative breast cancer patients. Additionally, the baseline circulating S1P levels were measured from samples taken from the patients after the removal of the tumor, hindering our ability to draw conclusions regarding the association of circulating S1P with the characteristics of breast cancer. Due to the initial scope of the study, follow-up data on outcomes is not available to dissect whether changes in S1P during chemotherapy were associated with differential recurrence or survival outcome.

## 5. Conclusion

In summary, this study displays a paradoxical association of low S1P with more aggressive subtypes, suggesting that circulating S1P may not be an accurate surrogate for tissue S1P in the tumor microenvironment. These data also reveal a variable association between various chemotherapy regimens and S1P, suggesting that S1P modulation could have a therapeutic impact in chemosensitization in breast cancer treatment.

## Figures and Tables

**Table 1:** Patient characteristics.

	Breast cancer patients (*n* = 158)	Control (*n* = 20)	*p* value
Age, years (SD)	51.2 (9.8)	51.3 (6.9)	0.99
BMI, kg/m^2^ (SD)	30.3 (7.6)	29.1 (7.4)	0.49
Ethnicity (%)			0.94
Caucasian African American Hispanic Other	92 (58.2) 53 (33.5) 6 (3.8) 7 (4.4)	13 (65.0) 6 (30.0) 1 (5.0) 0	
Tobacco use (%)	30 (19.0)	1 (5.0)	0.12
Alcohol use (%)	81 (51.2)	15 (75.0)	0.045
Postmenopausal (%)	89 (56.3)	11 (55.0)	0.91
Mean S1P, pmol/mL (SD)	1221.7 (439.4)	1139 (259.7)	0.412

## References

[1] Siegel R. L., Miller K. D., Jemal A. (2017). Cancer statistics, 2017. *CA: a Cancer Journal for Clinicians*.

[2] Takabe K., Kim R. H., Allegood J. C. (2010). Estradiol induces export of sphingosine 1-phosphate from breast cancer cells via ABCC1 and ABCG2. *The Journal of Biological Chemistry*.

[3] Takabe K., Spiegel S. (2014). Export of sphingosine-1-phosphate and cancer progression. *Journal of Lipid Research*.

[4] Takabe K., Paugh S. W., Milstien S., Spiegel S. (2008). “Inside-out” signaling of sphingosine-1-phosphate: therapeutic targets. *Pharmacological Reviews*.

[5] Takabe K., Yamada A., Rashid O. M. (2012). Twofer anti-vascular therapy targeting sphingosine-1-phosphate for breast cancer. *Gland Surgery*.

[6] Aoyagi T., Nagahashi M., Yamada A., Takabe K. (2012). The role of sphingosine-1-phosphate in breast cancer tumor-induced lymphangiogenesis. *Lymphatic Research and Biology*.

[7] Huang W. C., Nagahashi M., Terracina K. P., Takabe K. (2013). Emerging role of sphingosine-1-phosphate in inflammation, cancer, and lymphangiogenesis. *Biomolecules*.

[8] Mukhopadhyay P., Ramanathan R., Takabe K. (2015). S1P promotes breast cancer progression by angiogenesis and lymphangiogenesis. *Breast Cancer Management*.

[9] Nagahashi M., Yamada A., Miyazaki H. (2016). Interstitial fluid sphingosine-1-phosphate in murine mammary gland and cancer and human breast tissue and cancer determined by novel methods. *Journal of Mammary Gland Biology and Neoplasia*.

[10] Nagahashi M., Yamada A., Aoyagi T. (2016). Sphingosine-1-phosphate in the lymphatic fluid determined by novel methods. *Heliyon*.

[11] Nagahashi M., Ramachandran S., Kim E. Y. (2012). Sphingosine-1-phosphate produced by sphingosine kinase 1 promotes breast cancer progression by stimulating angiogenesis and lymphangiogenesis. *Cancer Research*.

[12] Nagahashi M., Kim E. Y., Yamada A. (2013). Spns2, a transporter of phosphorylated sphingoid bases, regulates their blood and lymph levels, and the lymphatic network. *The FASEB Journal*.

[13] Nagahashi M., Tsuchida J., Moro K. (2016). High levels of sphingolipids in human breast cancer. *The Journal of Surgical Research*.

[14] Tsuchida J., Nagahashi M., Nakajima M. (2016). Breast cancer sphingosine-1-phosphate is associated with phospho-sphingosine kinase 1 and lymphatic metastasis. *The Journal of Surgical Research*.

[15] Maiti A., Takabe K., Hait N. C. (2017). Metastatic triple-negative breast cancer is dependent on SphKs/S1P signaling for growth and survival. *Cellular Signalling*.

[16] Aoki M., Aoki H., Ramanathan R., Hait N. C., Takabe K. (2016). Sphingosine-1-phosphate signaling in immune cells and inflammation: roles and therapeutic potential. *Mediators of Inflammation*.

[17] Aoki M., Aoki H., Ramanathan R., Hait N. C., Takabe K. (2016). Corrigendum to “sphingosine-1-phosphate signaling in immune cells and inflammation: roles and therapeutic potential”. *Mediators of Inflammation*.

[18] Huang W. C., Liang J., Nagahashi M. (2016). Sphingosine-1-phosphate phosphatase 2 promotes disruption of mucosal integrity, and contributes to ulcerative colitis in mice and humans. *The FASEB Journal*.

[19] Donoviel M. S., Hait N. C., Ramachandran S. (2015). Spinster 2, a sphingosine-1-phosphate transporter, plays a critical role in inflammatory and autoimmune diseases. *The FASEB Journal*.

[20] Aoki H., Aoki M., Katsuta E. (2016). Host sphingosine kinase 1 worsens pancreatic cancer peritoneal carcinomatosis. *The Journal of Surgical Research*.

[21] Liang J., Nagahashi M., Kim E. Y. (2013). Sphingosine-1-phosphate links persistent STAT3 activation, chronic intestinal inflammation, and development of colitis-associated cancer. *Cancer Cell*.

[22] Lewis N. D., Haxhinasto S. A., Anderson S. M. (2013). Circulating monocytes are reduced by sphingosine-1-phosphate receptor modulators independently of S1P3. *Journal of Immunology*.

[23] Maeda Y., Matsuyuki H., Shimano K., Kataoka H., Sugahara K., Chiba K. (2007). Migration of CD4 T cells and dendritic cells toward sphingosine 1-phosphate (S1P) is mediated by different receptor subtypes: S1P regulates the functions of murine mature dendritic cells via S1P receptor type 3. *Journal of Immunology*.

[24] Singer I. I., Tian M., Wickham L. A. (2005). Sphingosine-1-phosphate agonists increase macrophage homing, lymphocyte contacts, and endothelial junctional complex formation in murine lymph nodes. *Journal of Immunology*.

[25] Guo S., Yu Y., Zhang N. (2014). Higher level of plasma bioactive molecule sphingosine 1-phosphate in women is associated with estrogen. *Biochimica et Biophysica Acta*.

[26] Sukocheva O., Wang L., Verrier E., Vadas M. A., Xia P. (2009). Restoring endocrine response in breast cancer cells by inhibition of the sphingosine kinase-1 signaling pathway. *Endocrinology*.

[27] Li J., Song Z., Wang Y. (2016). Overexpression of SphK1 enhances cell proliferation and invasion in triple-negative breast cancer via the PI3K/AKT signaling pathway. *Tumour Biology*.

[28] Maceyka M., Spiegel S. (2014). Sphingolipid metabolites in inflammatory disease. *Nature*.

[29] Navarro-Alvarez N., Soto-Gutierrez A., Chen Y. (2010). Intramuscular transplantation of engineered hepatic tissue constructs corrects acute and chronic liver failure in mice. *Journal of Hepatology*.

[30] Ghosal P., Sukocheva O. A., Wang T., Mayne G. C., Watson D. I., Hussey D. J. (2016). Effects of chemotherapy agents on sphingosine-1-phosphate receptors expression in MCF-7 mammary cancer cells. *Biomedicine & Pharmacotherapy*.

[31] Matula K., Collie-Duguid E., Murray G. (2015). Regulation of cellular sphingosine-1-phosphate by sphingosine kinase 1 and sphingosine-1-phopshate lyase determines chemotherapy resistance in gastroesophageal cancer. *BMC Cancer*.

[32] Ishitsuka A., Fujine E., Mizutani Y. (2014). FTY720 and cisplatin synergistically induce the death of cisplatin-resistant melanoma cells through the downregulation of the PI3K pathway and the decrease in epidermal growth factor receptor expression. *International Journal of Molecular Medicine*.

[33] Ihlefeld K., Vienken H., Claas R. F. (2015). Upregulation of ABC transporters contributes to chemoresistance of sphingosine 1-phosphate lyase-deficient fibroblasts. *Journal of Lipid Research*.

[34] Gstalder C., Ader I., Cuvillier O. (2016). FTY720 (fingolimod) inhibits HIF1 and HIF2 signaling, promotes vascular remodeling, and chemosensitizes in renal cell carcinoma animal model. *Molecular Cancer Therapeutics*.

[35] Ader I., Gstalder C., Bouquerel P. (2015). Neutralizing S1P inhibits intratumoral hypoxia, induces vascular remodelling and sensitizes to chemotherapy in prostate cancer. *Oncotarget*.

[36] Pchejetski D., Nunes J., Sauer L. (2010). Circulating sphingosine-1-phosphate inversely correlates with chemotherapy-induced weight gain during early breast cancer. *Breast Cancer Research and Treatment*.

